# Small Circular DNA Molecules as Triangular Scaffolds for the Growth of 3D Single Crystals

**DOI:** 10.3390/biom10060814

**Published:** 2020-05-26

**Authors:** Yu Wang, Xin Guo, Bo Kou, Ling Zhang, Shou-Jun Xiao

**Affiliations:** 1 State Key Laboratory of Coordination Chemistry, School of Chemistry and Chemical Engineering, Nanjing University, Nanjing 210093, China; yuwang0126@smail.nju.edu.cn (Y.W.); guoxin@smail.nju.edu.cn (X.G.); lingzhang@smail.nju.edu.cn (L.Z.); 2 School of Materials Science and Engineering, Nanjing Institute of Technology, Nanjing 211167, China; koubo@njit.edu.cn

**Keywords:** small circular DNA molecules, self-assembly, 3D DNA single crystals, triplex-forming oligonucleotides

## Abstract

DNA is a very useful molecule for the programmed self-assembly of 3D (three dimension) nanoscale structures. The organised 3D DNA assemblies and crystals enable scientists to conduct studies for many applications such as enzymatic catalysis, biological immune analysis and photoactivity. The first self-assembled 3D DNA single crystal was reported by Seeman and his colleagues, based on a rigid triangle tile with the tile side length of two turns. Till today, successful designs of 3D single crystals by means of programmed self-assembly are countable, and still remain as the most challenging task in DNA nanotechnology, due to the highly constrained conditions for rigid tiles and precise packing. We reported here the use of small circular DNA molecules instead of linear ones as the core triangle scaffold to grow 3D single crystals. Several crystallisation parameters were screened, DNA concentration, incubation time, water-vapour exchange speed, and pH of the sampling buffer. Several kinds of DNA single crystals with different morphologies were achieved in macroscale. The crystals can provide internal porosities for hosting guest molecules of Cy3 and Cy5 labelled triplex-forming oligonucleotides (TFOs). Success of small circular DNA molecules in self-assembling 3D single crystals encourages their use in DNA nanotechnology regarding the advantage of rigidity, stability, and flexibility of circular tiles.

## 1. Introduction

DNA self-assembling nanostructures are programmable, porous and scalable frameworks. The modular architecture of DNA molecules enables scientists to construct custom-shaped objects on the nanometer scale. In recent years, DNA tile bricks [1,2] and DNA origami [3,4,5,6,7] are the two most crucial and most commonly used bottom-up assembly technologies to design the subject-object system, in which DNA subject units as frameworks build up observable 3D (three dimension) crystalline materials, and object molecules of water, enzymes, and gold nanoparticles fill in framework pores. The advantage of tunable DNA nanostructure properties such as linking site, porosity, lattice geometry can be designed to conform the shape, volume and surface characteristics of target objects for applications in various research areas: catalysis, biological immune analysis, photoactivity, etc. [8,9,10,11,12,13,14]. The first structural DNA 3D crystal [15] was designed and achieved using branched DNA junctions to construct stacked triangular lattices, in which every two adjacent duplexes adopt approximately 60° in 3D and the side length of the triangle tile is two turns. Then the triangle tile side was extended into three turns, and the resulting crystals as subjects provided adequate geometric spaces to contain other molecular objects, which can be applied as special nanodevices [16,17]. With increasing the side length of triangle tiles, it is rational to infer that the triangle framework will become less rigid. To veneer the triangle framework, triplex-forming oligonucleotides (TFOs) have been applied to reinforce the tiles and crystals. TFOs offer the advantage that they post-anneal to the 3D crystal without interfering the crystallisation process and are compatible with a wide variety of oligonucleotide modifications, which introduce new functions and properties to the DNA crystal [18,19,20,21,22,23]. Later on, a series of comprehensive experimental conditions have been conducted toward crystallising larger multichain DNA objects [24,25,26]. The most recently reported DNA crystals with post-ligation can stand for a higher temperature up to 60 ℃, stepping forward to practical applications [27]. Compared to the diversified 2D and 3D arrays, successful designs of DNA 3D single crystals are countable, due to the challenging issues of perfect match and expansion of tiles in 3D space, and of which the tile should be both flexible to fit the joining gaps and rigid enough to fix the framework for crystallisation. We found out that the circular tile was much sturdier than the linear one with the same set of sequences for 2D nanostructures [28,29,30,31]. Herein, we tried on using the circular DNA molecule as the core triangle scaffold to build the triangular tile with three sides of the same length of three turns and further to grow 3D crystals. 3D DNA crystals in the size range from 10 to 450 µm were achieved by optimisation of crystallisation conditions and tuning of DNA sequences.

## 2. Materials and Methods

DNA sequences were designed according to [15] using the program SEQUIN. The sequences of the samples were noted in Supplementary Materials. 

### 2.1. Materials and DNA Strands

All DNA strands including Cy5 and Cy3 modified ones were bought from Sangon (Shanghai, China, www.sangon.com), which were purified by denaturing polyacrylamide gel electrophoresis (PAGE). Strands were dissolved at a concentration of approximately 300 μM in nuclease free water. Actual strand concentrations were determined by UV/vis detection of DNA absorbance at 260 nm. The enzymes T4 DNA ligase and exonuclease I were purchased from Takara Biotechnology Co., Ltd. (Dalian, China). Water (18 MΩ cm) was from a Milli-Q Ultrapure Water Purification System (Merck Millipore, Shanghai, China). Ethylenediaminetetraacetate (EDTA), urea, magnesium acetate tetrahydrate, boric acid, acetic acid, and tris(hydroxymethyl)aminomethane (Tris) were purchased from Sigma Aldrich Corp. (Shanghai, China). The TBE buffer is composed of 89 mM Tris, 89 mM boric acid, and 2 mM EDTA at pH 8.3, and the TAE-Mg buffer is composed of 40 mM Tris, 2 mM EDTA and 12.5 mM Mg(Ac)_2_ at pH 6.0 ± 0.5.

### 2.2. Preparation of Circular DNA

The circular DNAs of 48, 51, and 54 nt were circularised by T4 DNA ligase [28]. Firstly, a 5′-phosphorylated DNA strand (3.5 µM) and its corresponding 20 nt splint strand (4.5 µM) were mixed in 80 µL TE buffer (10 mM Tris-HCl, 1mM EDTA, pH = 8.0). The sample was heated to 95 °C for 5 min, then cooled down to room temperature within 4 h. The T4 ligase (350 U/μL, 10 μL) of 10 µL and 10× T4 buffer of 10 µL were added to the sample, then the mixture was incubated for 16 h at 16 °C. After reaction, the T4 ligase was inactivated at 95 °C for 5 min. Then, 10 µL 10× exonuclease I buffer and 10 µL exonuclease I (5 U/µL) were added to digest the remaining linear DNA residues of templates and splints by incubation at 37 °C for 30 min. The exonuclease I selectively digested the single-stranded DNA, and left the circular DNA intact. The circular DNA strands were purified by denaturing PAGE.

### 2.3. Crystallisation

The crystallisation procedure is consisted of two steps. In the first step of pre-crystallisation, a set of strands for each crystal were mixed at stoichiometric ratios, in 25 mM Tris-HCl and 12.5 mM MgCl_2_, pH = 8.0, with a concentration of the circular strand at 6 or 8 μM. The mixtures were annealed at a thermal ramp decreasing from 95 to 20 °C with 1 min/°C. In the second step of sitting-drop crystallisation, crystals were grown in a cryostat at 4 °C from a 5 µl sitting-drop containing 30 mM sodium cacodylate, 50 mM magnesium acetate, 50 mM ammonium sulfate, 5 mM magnesium chloride and 25 mM Tris-HCl (pH 8.5), against a reservoir solution containing 1.6 M ammonium sulfate for water vapour absorption. Crystals were detected with an LV100N POL polarising microscope (Nikon, Tokyo, Japan). In the post-annealing process of TFO crystals, each TFO strand was added twice of its stoichiometric amount.

### 2.4. TEM Imaging 

Crushed DNA crystals were prepared by adding 5 μL TAE-Mg buffer to the crystal drop and crushing the crystals into small pieces by cryo-loop under a microscope. A drop of 5 μL crushed crystals was then laid on a glow discharged TEM grid (Formvar/Carbon 400 mesh Cu grid) and stayed for 5 min for crystal attachment. Then, the grid was blotted by filter paper and stained by 2 μL 0.8% uranium acetate for 5 min. After rinsing in water, the grid was blotted and dried in air, and observed on a JEM-2100 transmission electron microscope (Japan Electronics Co., Ltd, Akishima, Japan).

### 2.5. Overall Crystal Designs

In this report, we crystallised six kinds of self-assembled DNA crystals listed in Table 1. Following the triangle tensegrity strategy, each triangular tile of six crystals was designed with nearly the same side length of three-turns but each tile has its own character. 

**Figure 1:**
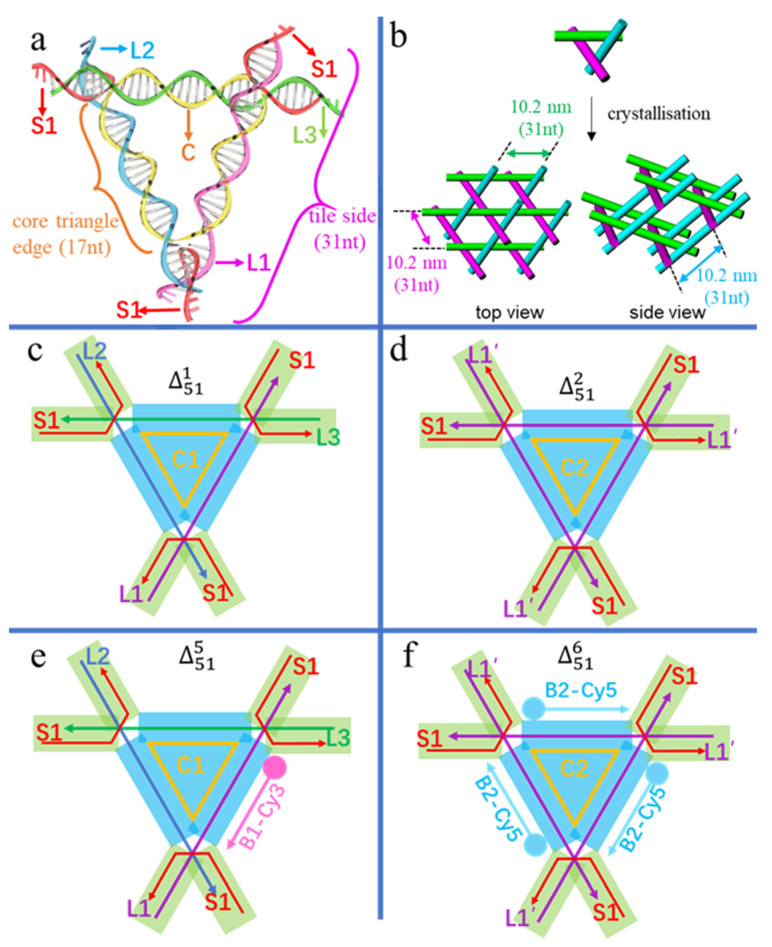
(**a**) The double helix model of the tensegrity triangular tile $\Delta_{51}^{n}$ (*n* = 1,2,5,6) with a circular core scaffold (C= C1 or C2) of three equal-length edges, three side helpers (L1, L2, L3) with the same length of three-turns, and three kinked helpers (S1) of the same sequence. The core triangle edge (17 nt/ bp) and tile side (31 nt/ bp) are distinguished as indicated. (**b**) Top and side views of tile stacking to 3D single crystals, with each cylinder representing a double helix. (**c**–**f**) Straight line models of individual triangular tiles with blue areas denoting the core triangle scaffold and green areas indicating six connection overhangs. (**c**) $\Delta_{51}^{1}$ of three differently-sequenced edges; (**d**) $\Delta_{51}^{2}$ of three-fold repeating sequences as three identical edges; (**e**) $\Delta_{51}^{5}$ of a modified $\Delta_{51}^{1}$ with an additional veneer TFO strand tagged with Cy3 (B1-Cy3) surrounding L1; (**f**) $\Delta_{51}^{6}$ of a modified $\Delta_{51}^{2}$ with three additional veneer Cy5-tagged TFO strands (3 × B2-Cy5).

To investigate the tolerance and perturbance of side length and sequence symmetry of the triangular tile, we modified the core triangle (scaffold) edge (Figure 1a) from the standard twist (17 bp) to an untwist (16 bp), and an overtwist (18 bp), as well as a three-fold repeating sequence for three symmetric edges and a non-repeating sequence for three asymmetric edges. Overall, the 3D circular triangular tile, which is labelled as $\Delta_{m}^{n}$ (m = the size of the circular DNA, n = the sequential number of crystals), contains three types of single-stranded DNAs in Figure 1a: a circular scaffold (yellow circular C), three tile side helpers (purple L1, blue L2 and green L3) with nearly the same length forming three-turns, and three kinked helpers (red polyline S1) that form three four-way junctions at the corners of the core triangle and have 2 nt sticky ends for specific stacking during crystallisation.

Association of these strands constructs two functional parts in the triangular tile of Figure 1c–f: a scaffold part of the core triangle (the blue areas in Figure 1c–f) and connection parts of the overhangs (the green areas in Figure 1c–f). The core triangle with three helix edges of equal length stretches into six overhangs slightly deviating from the triangular plane and yielding a 3D periodic lattice (Figure 1b). In detail, $\Delta_{51}^{1}$ (Figure 1c) contains a sequence-asymmetric circular core strand C1 of 51 nt, forming three equal-length edges of 17 bp. Moreover, each side of L1, L2 and L3 contains 31 nt, capable of forming three turns, and three S1 strands are 14 nt long. Denoted by symbols, $\Delta_{51}^{1}$ is made up of five components of C1, L1, L2, L3, and 3 × S1 in Table 1. It is obvious that three-fold repeats in the circular core triangle scaffold C2 in Figure 1d and f will have much higher spatial symmetry than the asymmetric C1 and will expand to perfect macroscopic 3D crystals. Denoted by symbols in Table 1, $\Delta_{51}^{2}$ is designed with three identical tile sides (3 × L1′) and three kinked helpers (3 × S1). The sequence of L1′ and thus of C2 are different from that of L1, or L2, or L3 and thus of C1, respectively. Likewise, $\Delta_{48}^{3}$ and $\Delta_{54}^{4}$ are two tiles altering the three repeating triangle edges into the length of 16 nt and 18 nt, respectively. Namely, circular 48 and 54 nt scaffolds with three-fold repeating sequences are utilised in $\Delta_{48}^{3}$ and $\Delta_{54}^{4}$, respectively. To examine the subject-object interactions, the L1 edge of $\Delta_{51}^{1}$ and the three identical L1′ edges of $\Delta_{51}^{2}$, were designed to bind to their third veneer TFO strands by Hoogsteen base pairing, resulting in two TFO-modified tiles of $\Delta_{51}^{5}$ in Figure 1e and $\Delta_{51}^{6}$ in Figure 1f. The TFO strand surrounding L1 in $\Delta_{51}^{5}$ was labelled with Cy3 (B1-Cy3), while the three TFOs surrounding L1′ in $\Delta_{51}^{6}$ were tagged with Cy5 (B2-Cy5). All six designs are listed in Table 1 and four of them are schematically illustrated in Figure 1.

## 3. Results and Discussion

The standard crystallisation protocol was as follows: all ingredients of a triangular tile with stoichiometric ratios were added together and mixed at 6 µM (if the influence of concentration was not explored), after pre-crystallisation from 95 to 20 °C at 1 min/°C, the drop-sitting crystallisation was proceeded via vapor exchange equilibration between the sample droplet and the buffer reservoir composed of 1.6 M (NH_4_)_2_SO_4_ for 6 days, if not otherwise noted. In some cases, the silicon oil was layered over the reservoir buffer to attain the desired crystals. The crystal cryostat was incubated at 4 °C to detectable crystals from 1 to 60 days via polarising microscope imaging. In general, to evaluate the stability of the four basic triangular tiles of $\Delta_{51}^{1}$, $\Delta_{51}^{2}$, $\Delta_{48}^{3}$ and $\Delta_{54}^{4}$, we analysed their gel-electrophoretic mobilities (Figure S1 of Supplementary Materials), in which each clear band of the four tiles indicated a size level at about 100 bp, similar to the theoretical position (93 bp) of $\Delta_{51}^{1}$ or $\Delta_{51}^{2}$. 

**Figure 2:**
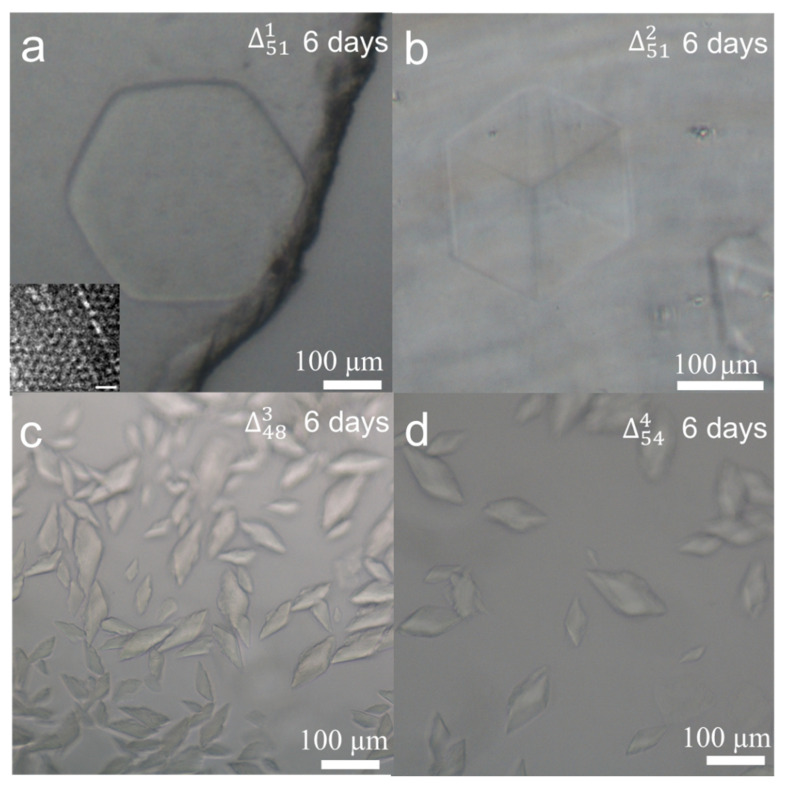
(**a**) A hexagonal crystal of $\Delta_{51}^{1}$ and an inset of its negatively stained TEM image with the scale bar of 20 nm, (**b**) a cubic crystal of $\Delta_{51}^{2}$, (**c**) small crystals of $\Delta_{48}^{3}$, (**d**) small crystals of $\Delta_{54}^{4}$. All crystals were grown at 6 µM for 6 days.

To optimise the ingredient concentration and incubation time for crystallisation, we took $\Delta_{51}^{1}$ as an example. A series of DNA concentrations from 1 to 10 µM were prepared and hexagonal crystal tablets could be observed from 4 to 10 µM solutions. After evaluation, we chose the DNA concentration of 6 µM as the standard for all samples because it could produce several big crystals but not many small crystal seeds. Normally, DNA crystal seeds could be easily detected after one day. Six days later, hexagonal crystals from $\Delta_{51}^{1}$ were observed in an edge size of two hundred microns in Figure 2a and more crystal photos were listed in Figure S2. For all crystallisation experiments, we set a time criterion of 6 days for optical imaging assay. To grow larger crystals, longer incubation time is needed. Approximately with 60 days’ incubation, larger hexagonal crystals with an edge size of about 450 µm appeared (Figure S3). To investigate the crystal’s microstructure, the crystal was micropipetted into small pieces and negatively stained with uranyl acetate for TEM (transmission electron microscopy) imaging. From the theoretical models of Figure 1b, hexagonal and quadrilateral lattice patterns can be expected by top and side views. In the inset of Figure 2a and in Figure S4, the measured lattice constant of 10.0 nm and the distance of 9.0 nm between two parallel helixes are in line with theoretical estimation. Due to the random breaking of microcrystals, different lattice patterns were observed by TEM imaging from different crystal orientations.

To analyse the effect of sequence and length of the core triangle scaffold on the 3D crystal quality, three modifications were conducted. The first modification is that the circular 51 nt scaffold is composed of three-fold repeating sequences, resulting in $\Delta_{51}^{2}$. Compared to the hexagonal crystal plates of $\Delta_{51}^{1}$, the crystal shape changed into cubes (Figure 2b) and more crystal photos were shown in Figure S5. In theory, $\Delta_{51}^{2}$ owns a triad rotation symmetry (L^3^), which is higher in spatial symmetry than the asymmetric $\Delta_{51}^{1}$. In macroscale, the cube of $\Delta_{51}^{2}$ with higher symmetry in Figure 2b occupies a volume of ~120 × 120 × 120 μm^3^, about twice of the hexagonal plate of $\Delta_{51}^{1}$ (~$\frac{3\sqrt{3}}{2}$ × 120 × 120 × 20 μm^3^) in Figure 2a. To evaluate the tolerance of the core triangle edge length, we modify the edge to decrease or increase 1 bp, resulting in $\Delta_{48}^{3}$ and $\Delta_{54}^{4}$. Under the standard concentration of 6 μM and incubation time of 6 days, many irregular and much smaller crystals were observed with the size at about 70 µm in Figure 2c,d. As the tile side length of $\Delta_{51}^{2}$ is 31 (17 + 14) bp, $\Delta_{48}^{3}$ is 30 (16 + 14) bp and $\Delta_{54}^{4}$ is 32 (18 + 14) bp, all closing to three full turns, we suggest that the small deviations of six overhangs from the core triangle plane cause the crystal structural changes. To sum, the stereo structure of $\Delta_{51}^{2}$ is perfect for crystal growth, whereas in $\Delta_{48}^{3}$ or $\Delta_{54}^{4}$, each overhang overtwists or untwists about ±34° from the reference $\Delta_{51}^{2}$, which may cause frustration, dislocation, and distortion of the crystal framework, and finally results in many small and irregular crystals.

**Figure 3:**
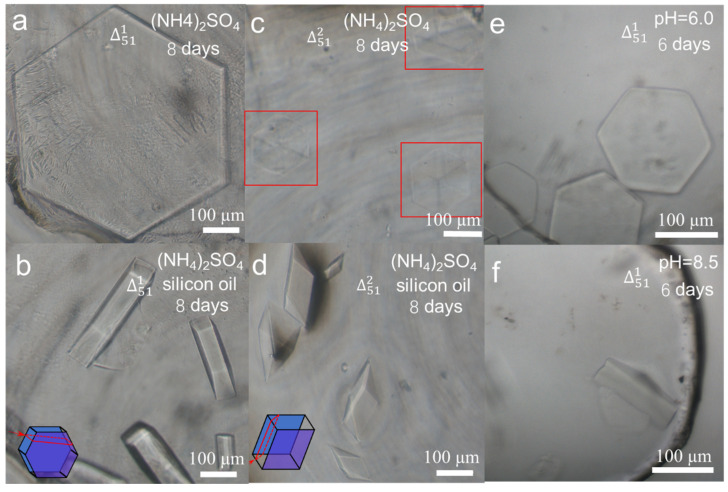
(**a**,**b**) Different crystal morphologies of $\Delta_{51}^{1}$ without and with silicon oil floating on the reservoir buffer. The schematic diagram at the left bottom corner of (**b**) shows that a hexagonal tablet can be cut into a prismatoid prism. (**c**,**d**) Different crystal morphologies of $\Delta_{51}^{2}$ without and with the silicon oil covering. The schematic diagram at the left bottom corner of (**d**) shows that a triangular prism can be cut from a cube. (**e**,**f**) Different crystal shapes of $\Delta_{51}^{1}$ at pH 6.0 and 8.5.

Except for the ingredient concentration and incubation time, other parameters in the crystallisation procedure, for example, water vapor diffusion speed between sampling buffer and reservoir buffer [32] and pH of sampling buffer [33,34,35], also influence the crystal qualities (Figures S2 and S5). Generally, plentiful tiny crystals were formed in the DNA crystal trials, instead of the bigger single crystals in the size of hundreds of microns, which were not qualified for inspection. In many cases, tiny crystals occurred quickly because the sampling solution was evaporated too fast, resulting in much higher sample concentration, and reaching supersaturation. Hence, effective reduction of the crystallisation speed was a way to acquire large crystals. Improvement [36] by setting a barrier at the vapour-diffusion interface between the reservoir buffer and the sampling drop could decrease the water vapor diffusion speed. The simplest way to control the diffusion speed was to place a fixed volume of silicon oil above the reservoir buffer of ammonium sulfate. Silicon oil is less dense than ammonium sulfate buffer and therefore the oil floats on the buffer of the reservoir (Figure S6). In this way, the crystal shapes of $\Delta_{51}^{1}$ were different from those without silicon oil. $\Delta_{51}^{1}$ and $\Delta_{51}^{2}$ were just like prismatoid (Figure 3b) and triangular prism (Figure 3d) that were parts of their mother hexagonal prisms (Figure 3a) and cuboids (Figure 3c), respectively. The schematic diagrams showing the part and whole relationship were attached into Figure 3b and d at their left bottom corners as inserts, separately. All the four crystals were incubated for 8 days, so the crystals in Figure 3a,c were bigger than their corresponding ones in Figure 2 for 6 days’growth. On the 3D scale, the crystals grew more intensive and bigger when the silicon oil was layered onto the reservoir buffer, thus the crystals can be easily conducted for inspection, data collection and processing, and structural determination [15,37]. In detail, the thickness of $\Delta_{51}^{1}$ changed from 20 µm to about 80 µm after layering silicon oil. Likewise, the thickness of $\Delta_{51}^{2}$ changed from 120 µm to about 150 µm. So far, we found that the oil coating method could slow down the water vapor diffusion speed, balance the DNA crystal growth along more directions, and decrease the crystal symmetry in 3D. In addition, pH was one of the most extensive triggers to affect DNA conformations and mechanics, and thus crystal morphologies and qualities [33,34,35]. We carried out crystallisation of $\Delta_{51}^{1}$ at pH ranging from 6.0 to 8.5 to explore the feasibility of growth and the stability of crystals. The crystals were captured at pH 6.0 (Figure 3e) and 8.5 (Figure 3f) of sampling buffers after 6 days’ incubation. At pH 6.0, the crystals were hexagonal plates with the side length of approximately 100 µm, while the crystals at pH 8.5 were prismatoids with the side length of 120 µm. We suggest that when pH changs from 6.0 to 8.5, charges of DNA strands change too, thus coulomb repulsions between DNA tiles, as well as between DNA molecules and the buffer environments differently. Such an environmental change slowed down the crystal growth towards the prismatic directions and promoted the growth along the basal directions. From the above experiments, we suggest that the oil covering method and the slightly alkaline sampling buffer will help the growth of DNA single crystals to larger sizes comparably in 3D, which could be favored for X-ray diffraction.

**Figure 4:**
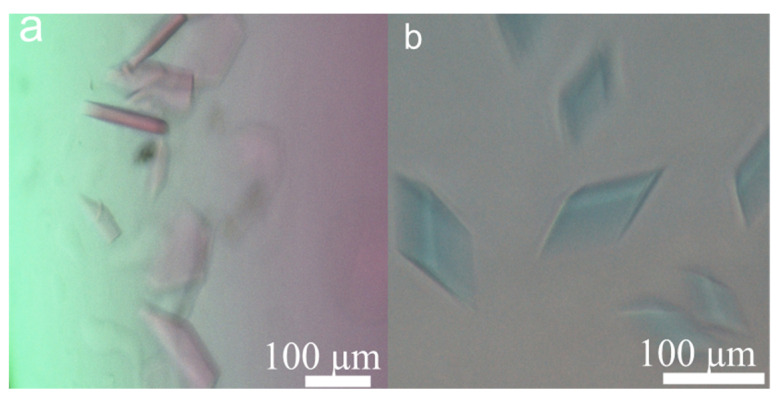
(**a**) Crystals of $\Delta_{51}^{5}$ stained with Cy3, (**b**) crystals of $\Delta_{51}^{6}$ stained with Cy5.

To explore whether the circular DNA crystals could be used as a subject system to host object molecules, two additional crystals were assembled as $\Delta_{51}^{5}$ and $\Delta_{51}^{6}$. Although both one-pot and post-objecting crystallisations could be carried out to achieve DNA crystals, we prefer the post-objecting method because the fluorescent labels on the third TFO strand may not match the crystal organisation in 3D space perfectly, and sometimes could not tolerate the water boiling temperature for annealing at pre-crystallisation and the slow crystallisation process. To avoid the experimental heterogeneity, the strategy by introducing triple helixes for DNA recognition [38] into the DNA crystal at 4 ℃ was adopted in this work. In this way, guest molecules, Cy3 and Cy5 labelled TFO strands, were incorporated at target locations of triangular tiles, separately (Figure 1e,f). Meanwhile, the fluorescence tracing experiments, in which the crystals can be fluorescently stained, also confirmed that the crystals came from DNA triangular tiles but not from inorganic salts. TFOs were added into the crystal buffer and incubated for weeks to obtain the corresponding crystals (Figure 4a,b). Control experiments of $\Delta_{51}^{1}$ crystals for specific binding of B1-Cy3 but not B2-Cy5 were carried out and the results were shown in Figure S7. Through the porous structure of triangular frameworks, TFOs diffused into their corresponding duplexes gradually. From the staining experiments, we suggest that water molecules in DNA crystals are not completely fixed, there should be at least two types of water molecules, solid icy and liquid water molecules. Icy water molecules help DNA frameworks solidifying the crystal, while liquid water molecules help object molecules diffusing into the crystal. Formation of triple helixes is through sequence-specific recognition (T-AT and C^+^-GC) that a TFO binds to the major groove of a duplex by generating Hoogsteen hydrogen bonds. The stained crystals were geometrically intact but crystal colours demonstrated that the 3D DNA crystals were endowed with new physical properties. Thus, when the third strand is modified with other functional species, such as enzymes, nanoparticles, and actuation species, many interesting functions will be generated.

## 4. Conclusions

In summary, we have demonstrated that the circular DNA as the core scaffold forming the triangular tile can be applied to construct 3D DNA single crystals. To obtain the suitable size of DNA crystals for optical microscope inspection, the concentration of triangular tiles was screened to be 6 µM and the time to be about 6 days, which were considered to be optimum for balancing between crystal quality and labor- and/or time-cost. Under the similar crystal growth conditions, crystals from circular DNAs (120 μm for 6 days) were bigger than those of the same design but with a whole set of linear DNAs (70 μm for 6 days) [16]. The biggest crystal size we achieved with circular triangular tiles is 450 μm of the regular hexagonal side in $\Delta_{51}^{1}$ single crystals. The hexagonal and cubic crystals here possess much higher symmetry, which demonstrate that the orientation angles among helices match the tile-based self-assembly for accurate lattice growth in 3D. The crystals grown with circular scaffolds can also provide internal porosities for hosting object molecules with different functions as in linear DNA crystals, for example, diffusion of Cy3 and Cy5 labelled TFO strands to colour the original and colourless $\Delta_{51}^{1}$ and $\Delta_{51}^{2}$ crystals. In addition to the usual crystallisation conditions, two extra parameters were tested to grow bigger crystals: layering oil onto the reservoir buffer and adjusting pH of the sampling buffer below and above the neutral state a little bit. In addition, for the triangular crystal framework of the tile side length of three turns, due to the spring-like structure of DNA molecules, a little flexibility in the helical periodicity still remains, for example, $\Delta_{48}^{3}$ and $\Delta_{54}^{4}$ which are deviated with 1 bp more or less from the regular three turns can still grow tiny crystals. It is rational to infer that a trivial twist on the level of single tiles will rapidly accumulate in 3D single crystals, and such deviation accumulation cannot be corrected if the tiles are too rigid, thus causes defections during the crystal growth and results in irregular and tiny crystals. Overall, successful growth of DNA single crystals with small circular DNA molecules as core triangular scaffolds indicates the use of small circular DNAs in DNA nanotechnology is not limited.

## Figures and Tables

**Table 1:** Six triangle tiles with strand components ^#^ and their 3D crystal shapes and estimated sizes *.

	$\Delta_{51}^{1}$	$\Delta_{51}^{2}$	$\Delta_{48}^{3}$	$\Delta_{54}^{4}$	$\Delta_{51}^{5}$	$\Delta_{51}^{6}$
Tile Components	C1, L1, L2, L3, 3 × S1	C2, 3×L1′, 3 × S1	C3, 3 × L4, 3 × S1	C4, 3 × L5, 3 × S1	C1, L1, L2, L3, 3 × S1, B1-Cy3	C2, 3 × L1′, 3 × S1, 3 × (B2-Cy5)
Crystal Shapes and Estimated Sizes	Hexagonal prism, a(= b) = 100–450 µm, c = 20–40 µm	Cube, a(= b = c) = 100–150 µm	Oblique triangular prism, a(= b) = 20~30 µm, c = 10~30 µm	Oblique triangular prism, a(= b) = 30~50 µm, c = 10~30 µm	Hexagonal prism, a(= b) = 100~150 µm, c = 10~20 µm	Oblique triangular prism, a(= b) = 60~110 µm, c = 40~60 µm

^#^ Tile and strand representation symbols please refer to Figure 1 and descriptions of this section; * 3D crystal shapes are roughly described according to their 3D geometries, and crystal sizes were measured and estimated by the ruler on the lens of the polarising microscope, assuming a(= b) represents the edge of a regular base polygon, and c the height of the crystal.

## Supplementary Materials

The following are available online at https://www.mdpi.com/2218-273X/10/6/814/s1, Figure S1: Native PAGE assay. Figure S2: More optical crystal photos of $\Delta_{51}^{1}$. Figure S3: The biggest crystals with the size indicated by the ruler of the polarising microscope. Figure S4: Negatively stained TEM images of $\Delta_{51}^{1}$ crystals. Figure S5: More optical crystal photos of $\Delta_{51}^{2}$. Figure S6: Schematic device for crystallisation with silicon oil floating on the reservoir buffer. Figure S7: Control experiments of $\Delta_{51}^{1}$ crystals for specific binding of Cy3-labelled TFOs.
