# Supplementary material for: Small Circular DNA Molecules as Triangular Scaffolds for the Growth of 3D Single Crystals
Source: Biomolecules. 2020 May 26;10(6):814. doi: 10.3390/biom10060814 (PMC7355631; doi:10.3390/biom10060814)
Supplement: Supplementary file 1 [file biomolecules-10-00814-s001.pdf]

biomolecules

Supplementary Materials

Small Circular DNA Molecules as Triangular Scaffolds for Growth of 3D Single Crystals

Yu Wang [1,†], Xin Guo [1,†], Bo Kou [2], Ling Zhang [1] and Shou-Jun Xiao [1,*]

1 State Key Laboratory of Coordination Chemistry, School of Chemistry and Chemical Engineering, Nanjing University, Nanjing 210093, Jiangsu, China; yuwang0126@smail.nju.edu.cn (Y.W.); guoxin@smail.nju.edu.cn (X.G.); lingzhang@smail.nju.edu.cn (L.Z.)
2 School of Materials Science and Engineering, Nanjing Institute of Technology, Nanjing, 211167, China; koubo@njit.edu.cn
* Correspondence: sjxiao@nju.edu.cn
† These authors contributed equally to this study.

This file includes:

1. DNA sequences used in this work
2. Native Polyacrylamide Gel Electrophoresis
3. Figure S1. Native PAGE assay
4. Figure S2. More optical crystal photos of Δ^1_{51}
5. Figure S3. The biggest crystals with the size indicated by the ruler of the polarising microscope
6. Figure S4. Negatively stained TEM images of Δ^1_{51} crystals
7. Figure S5. More optical crystal photos of Δ^2_{51}
8. Figure S6. Schematic device for crystallisation with silicon oil floating on the reservoir buffer
9. Figure S7. Control experiments of Δ^1_{51} crystals for specific binding of Cy3-labelled TFOs

1. DNA sequences used in this work

Δ^1_{51}: C1: 5’- CCATTCAGGCTGCGCAA
CTGTTGGGAAGGGCGAT
CGGTGCGGGCCTCTTCG-3’
L1: 5’-TCGCAGCCTTGCGCAGCCTGAATGGGGCTGC-3’
L2: 5’-TCGCAGCCATCGCCCTTCCCAACAGGGCTGC-3’
L3: 5’-TCGCAGCCCGAAGAGGCCCGCACCGGGCTGC-3’
S1: 5’-GAGCAGCCGGCTGC-3’ (the red codes are sticky ends)

Δ^2_{51}: C2: 5’ -GACAACCTAAGATTAGG
GACAACCTAAGATTAGG
GACAACCTAAGATTAGG-3’
L1′: 5’-TCGCAGCCCCTAATCTTAGGTTGTCGGCTGC-3’
S1: 5’-GAGCAGCCGGCTGC-3’ (the red codes are sticky ends)

Δ^{3}_{48}: C3: 5’ -GACAACCTAAGATTAG
GACAACCTAAGATTAG
GACAACCTAAGATTAG-3’
L4: 5’-TCGCAGCCCTAATCTTAGGTTGTCGGCTGC-3’
S1: 5’-GAGCAGCCGGCTGC-3’ (the red codes are sticky ends)

Δ^{4}_{54}: C4: 5’ -GACAACCTAAGATTAGGT
GACAACCTAAGATTAGGT
GACAACCTAAGATTAGGT-3’
L5: 5’-TCGCAGCCACCTAATCTTAGGTTGTCGGCTGC-3’
S1: 5’-GAGCAGCCGGCTGC-3’ (the red codes are sticky ends)

Δ^{5}_{51}: C1: 5’- CCATTCAGGCTGCGCAA
CTGTTGGGAAGGGCGAT
CGGTGCGGGCCTCTTCG-3’
L1: 5’-TCGCAGCCTTGCGCAGCCTGAATGGGGCTGC-3’
L2: 5’-TCGCAGCCATCGCCCTTCCCAACAGGGCTGC-3’
L3: 5’-TCGCAGCCCGAAGAGGCCCGCACCGGGCTGC-3’
S1: 5’-GAGCAGCCGGCTGC-3’ (the red codes are sticky ends)
B1-Cy3: Cy3-5’-CCTTTCTCCCTCCCCTT-3’

Δ^{6}_{51}: C2: 5’ -GACAACCTAAGATTAGG
GACAACCTAAGATTAGG
GACAACCTAAGATTAGG-3’
L1′: 5’-TCGCAGCCCCTAATCTTAGGTTGTCGGCTGC-3’
S1: 5’-GAGCAGCCGGCTGC-3’ (the red codes are sticky ends)
B2-Cy5: Cy5-5’- CTCTTCCTTTCTTTTCC-3’

2. Native Polyacrylamide Gel Electrophoresis

Each annealed sample was mixed with the same amount of Glycerol Gel Loading Buffer (0.25% Bromophenol Blue; 0.25% Xylene Cyanole FF; 60% Glycerol). Electrophoresis was carried out in 8% native polyacrylamide gel for 3 h at 90 V in TAE-Mg buffer. A low-molecular weight DNA ladder (10 mM Tris-HCl, pH 7.6, 1 mM EDTA, 0.033% bromophenol blue, 0.008% xylene cyanol and 10% glycerol) was added in the first lane as the migration standard (bands of 25, 50, 75, 100, 150, 200, 300, 400, and 500 bp from bottom to top). Dyed by 4S GelRed for 1 hour, the gels were scanned using a Tanon 2500R laser scanner to give a gel image in Figure. S1.

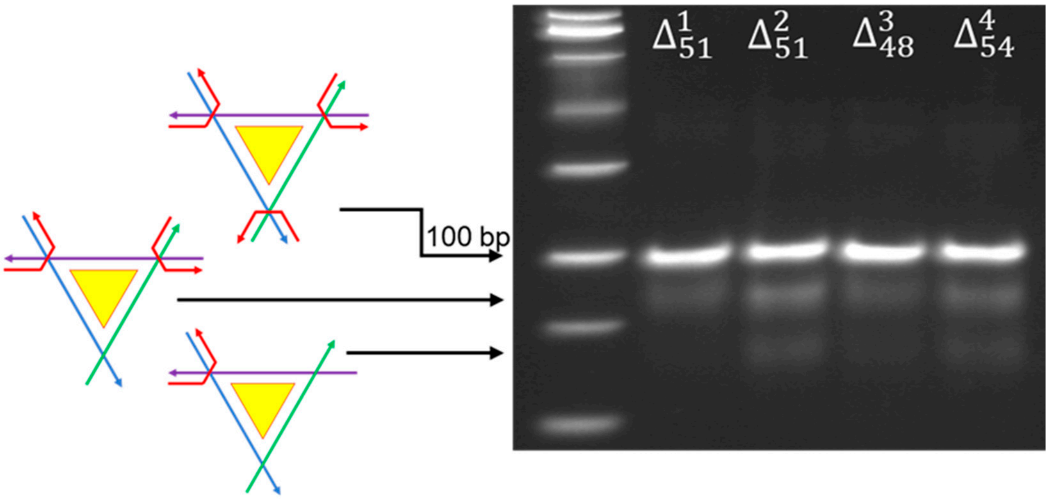

Figure S1. Native PAGE assay. Native polyacrylamide gel electrophoresis (PAGE) analysis of four triangle tiles of Δ^1_{51}, Δ^2_{51}, Δ^3_{48}, and Δ^4_{54} at 4 ℃. The tile symbol for each sample is indicated at the top of the gel lane and the chemical identity of each band is suggested at the left side. Apart from the sequence-asymmetric tile of Δ^1_{51} with each tile side having different sequences, each of other three triangle tiles of Δ^2_{51}, Δ^3_{48} and Δ^4_{54} has identical three tile sides, in which each circular scaffold has a three-fold repeating sequence. The schematic drawings at the left side represent the chemical identities of bands, separately. The meanings of schematic drawings please refer to the caption of Figure. 1 in the main text.

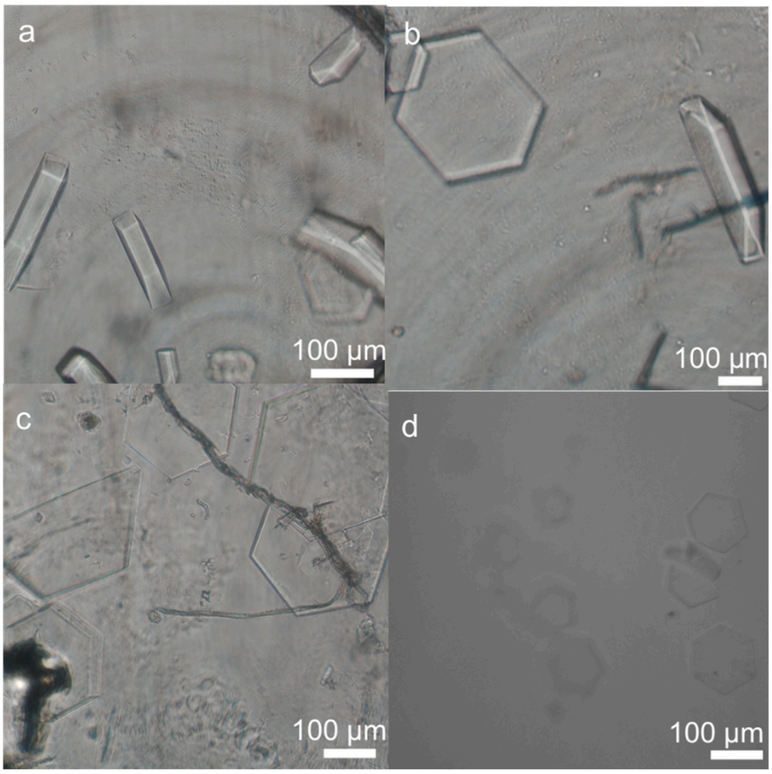

Figure S2. More optical crystal photos of $\boldsymbol{\Delta_{51}^{1}}$. (a, b) Crystals grown in a sitting drop with silicon oil floating on the reservoir buffer, (c, d) crystals grown without silicon oil, under the conditions of the triangular tile concentration at 6.0 µM and the incubation time of 6 to 8 days.

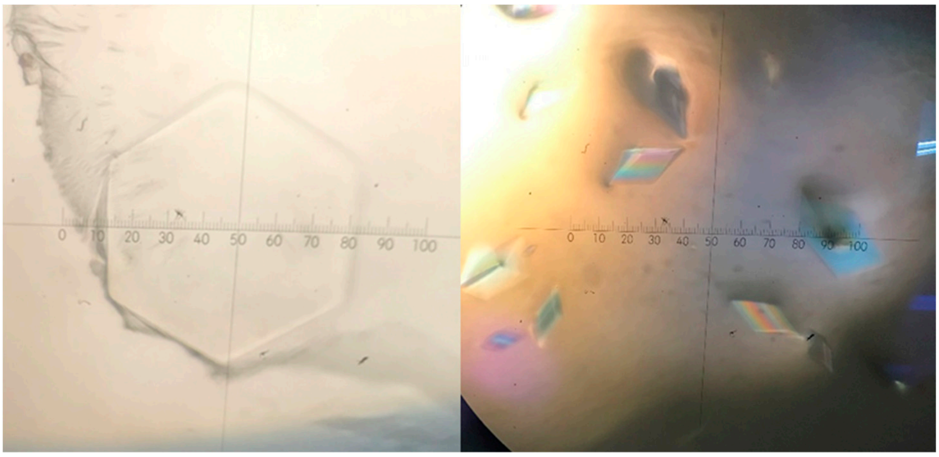

Figure S3. The biggest crystals with the size indicated by the ruler of the polarising microscope. The crystal sizes of $\boldsymbol{\Delta_{51}^{1}}$ and $\boldsymbol{\Delta_{51}^{2}}$ were recorded by the ruler on the lens (the smallest scale of the ruler is 10 µm). The biggest hexagonal side was measured at 450 µm and the biggest rhombic side at 160 µm, respectively. The crystallisation conditions were with the tile concentration at 10.0 µM and the incubation time over 60 days.

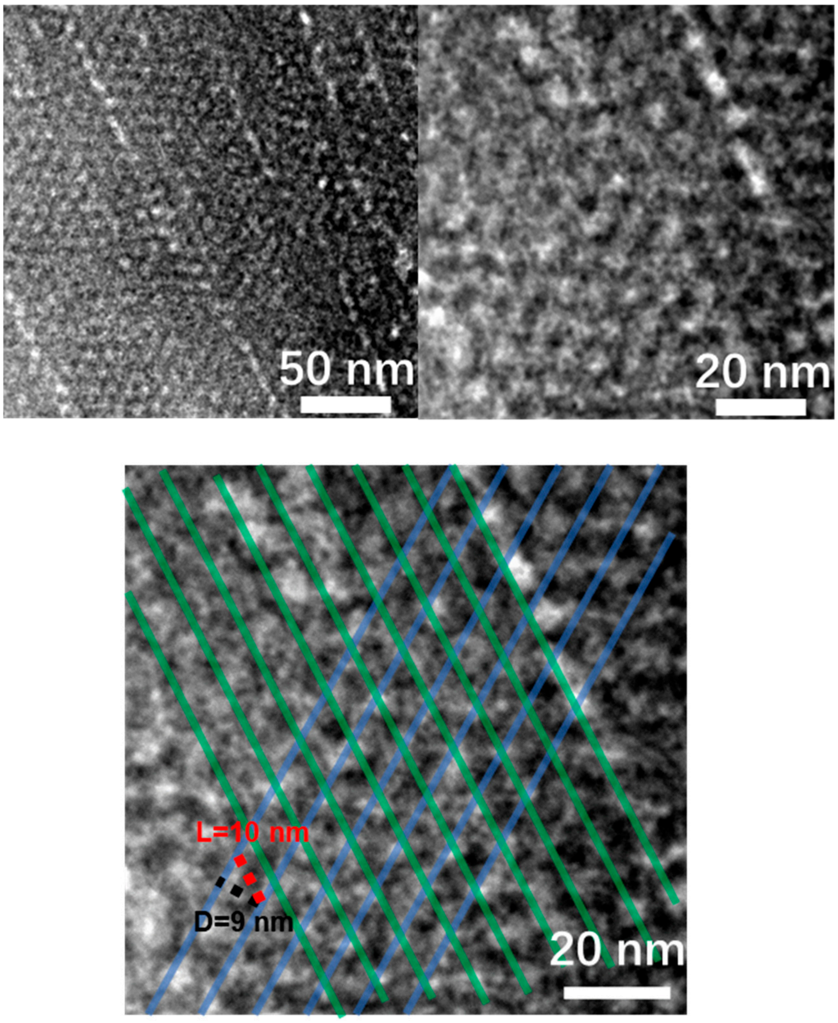

Figure S4. Negatively stained TEM images of Δ^1_{51} crystals. Microstructual lattices of Δ^1_{51} crystals in nanometer scale are shown, with rhombohedral lattice mapping at the bottom.

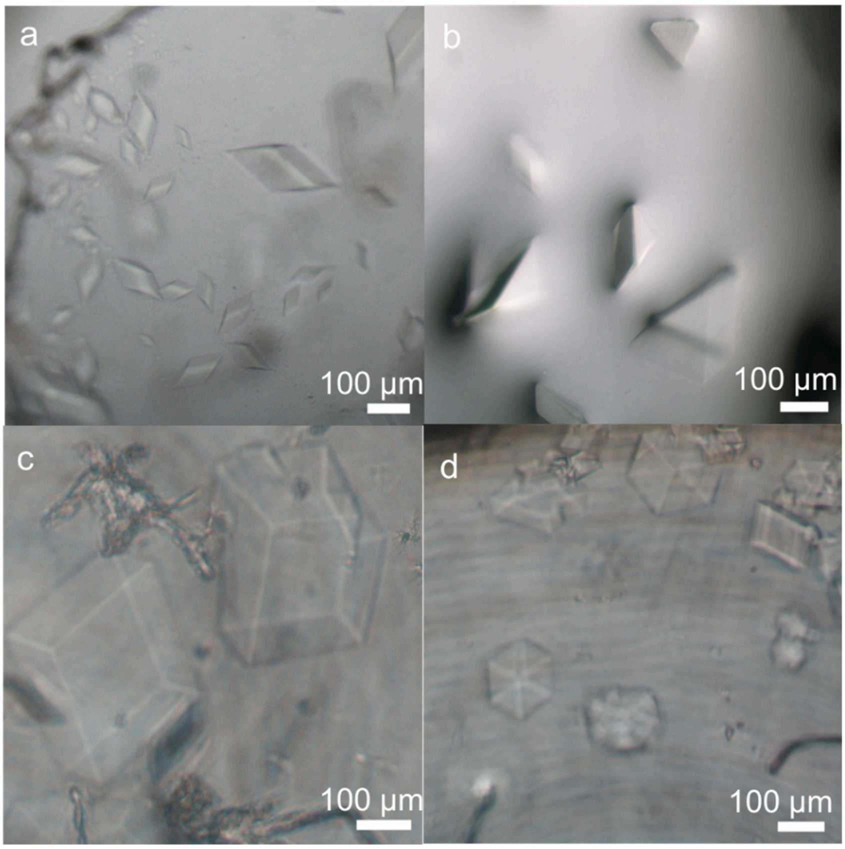

Figure S5. More optical crystal photos of Δ^2_{51}. (a, b) Crystals grown with silicon oil floating on the reservoir buffer, (c, d) crystals grown without silicon oil, under the conditions of the tile concentration at 6.0 μM and the incubation time of 6 to 8 days.

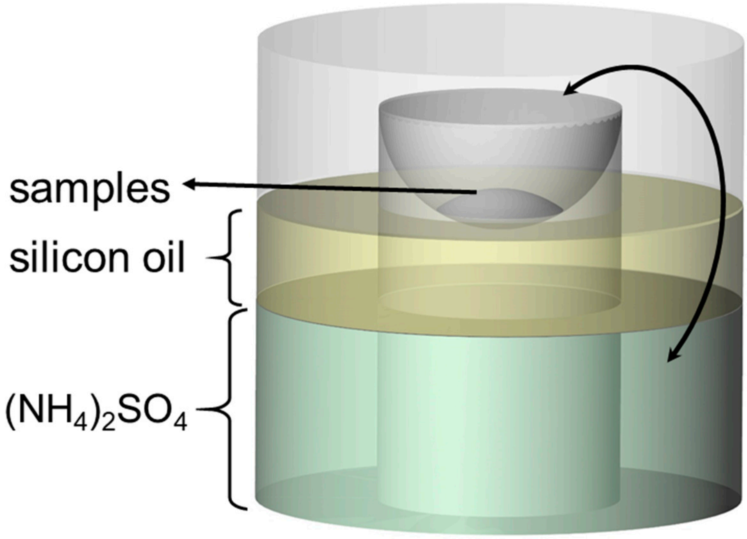

Figure S6. The schematic device for crystallisation with silicon oil floating on the reservoir buffer. The silicon oil (yellow) floating on the reservoir buffer formed a barrier to slow down the water vapour diffusion speed from the sample drop to the absorbing $(NH_4)_2SO4$ buffer.

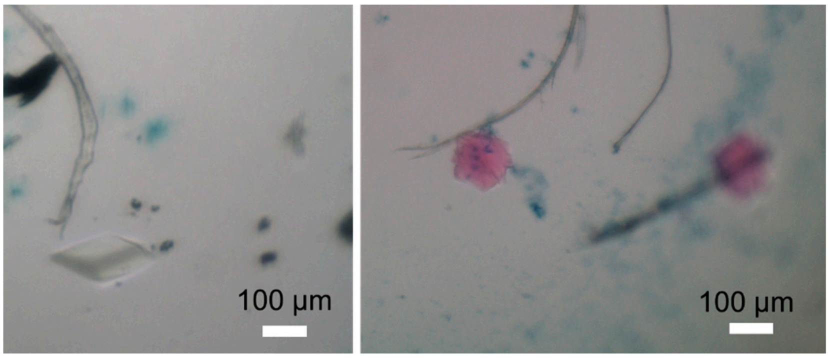

Figure S7. Control experiments of $\mathbf{\Delta^{1}_{51}}$ crystals for specific binding of Cy3-labelled TFOs. At the left, a $\mathbf{\Delta^{1}_{51}}$ crystal incubated with Cy5-labelled TFO B2-Cy5 (blue spots) remains transparent; at the right, two $\mathbf{\Delta^{1}_{51}}$ crystals incubated with both B2-Cy5 (blue regions) and Cy3-labelled TFO B1-Cy3 become red; which indicate that L1 of $\mathbf{\Delta^{1}_{51}}$ specifically binds B1-Cy3.

© 2020 by the authors. Licensee MDPI, Basel, Switzerland. This article is an open access article distributed under the terms and conditions of the Creative Commons Attribution (CC BY) license (http://creativecommons.org/licenses/by/4.0/).
